# Effects of Menstrual Cycle on Exercise Treadmill Parameters and Cardiac Troponin Release in Premenstrual Women

**DOI:** 10.3390/diagnostics15121548

**Published:** 2025-06-18

**Authors:** Aysu Oktay, Inanc Torustag, Ferruh Kemal Isman, Mehmet Agirbasli

**Affiliations:** 1 Department of Cardiology, Istanbul Medeniyet University, Istanbul 34722, Turkey; inanctorustag@saglik.gov.tr; 2 Department of Clinical Biochemistry, Istanbul Medeniyet University, Istanbul 34722, Turkey; ferruhisman@yahoo.com

**Keywords:** high-sensitivity troponin, menstrual cycle, ST/HR index

## Abstract

**Background:** The diagnostic accuracy of the exercise treadmill test (ETT) remains suboptimal in premenopausal women. Menstrual cycle phases display hormonal variations and biological effects in premenopausal women. The early and late follicular phases of the menstrual cycle demonstrate nearly four-fold differences in estrogen levels. **Methods:** This study assessed the variability in ETT results between the early and late follicular phases in premenopausal women. This study included premenopausal females with regular menstrual cycles and chest pain. As per the study protocol, patients underwent two separate ETTs at the early and late follicular phases of the menstrual cycle. Hormones and high-sensitivity cardiac troponin T (hs-cTnT) were measured. The primary endpoint was the ST segment/heart rate (HR) index. The secondary endpoints were maximum ST/HR slope, ST segment depression, HR and blood pressure (BP) response, exercise capacity, and hs-cTnT change after ETT. **Results:** False-positive ETT results were common in premenopausal women. The early follicular phase displayed significantly higher hs-cTnT and BP responses to ETT compared to the late follicular phase. This study reports that ETT results are similar between the early and late follicular phases of the menstrual cycle in premenopausal women. Biological variability is observed in the BP and hs-cTnT response to ETT between the two phases. **Conclusions:** The menstrual cycle phase (early versus late follicular phase) did not affect the ETT results. The consideration of estrogen and hormonal status when evaluating the diagnostic test results can improve our understanding of cardiovascular disease in women.

## 1. Introduction

Significant variability exists in the sensitivity (23–100%) and specificity (17–100%) of the exercise treadmill test (ETT) for detecting coronary artery disease (CAD) in women with low to intermediate risk for CAD [1,2,3]. False-positive stress ECG results are commonly observed in premenopausal women, which limits the specificity of the test [3,4,5]. The biology of false-positive ETT results in women is not well understood. False-positive ETT results often lead to unnecessary further diagnostic workups, exposing patients to radiation and contrast agents [6]. Therefore, understanding the potential causes of false-positive ETT results in premenopausal women is clinically important. Menstrual cycles cause periodic biological variability in premenopausal women that can affect ETT results [7]. The menstrual cycle consists of the early follicular (days 1–5), late follicular (days 6–12), ovulation (days 13–15), early luteal (days 16–19), mid-luteal (days 20–23), and late luteal (days 24–28) phases. During the menstrual cycle, estrogen levels can fluctuate nearly fourfold between the early and late follicular phases. The early follicular phase is characterized by low estrogen and low progesterone levels, whereas the late follicular phase is marked by high estrogen levels and low progesterone [7,8,9].

The effects of endogenous and exogenous estrogen on coronary ischemia are com-plex. Estrogen has direct and indirect consequences on atherosclerosis and ischemia. Experimental and clinical studies provide contradictory findings on the vascular effects of estrogen. Both the vasodilatory and vasoconstrictor effects of estrogen have been reported in thermoregulation and physical work capacity in women [9,10,11]. With the discovery of high-sensitivity cardiac troponins (hs-cTn), minute changes in hs-cTn have become detectable [12]. The causes and clinical significance of ETT-induced hs-cTn leak remain unknown. Similarly, the effects of estrogen on the biological variability of hs-cTnT remain to be elucidated. This study aims to identify the biological variation in ETT results between the early and late follicular phases of the menstrual cycle in premenopausal women.

## 2. Materials and Methods

This study was conducted in the cardiology clinics of a tertiary care training hospital. Ethical approval was obtained from the local ethics committee (approval number: 2023/0129, dated 22 February 2023). This study was registered at ClinicalTrials.gov (Protocol Record: 2023/0129, NCT05985980).

## 3. Study Population

The study population consisted of premenopausal women (20–55 years old) who presented to the outpatient clinic with chest pain between February 2023 and July 2023. All participants reported having a regular menstrual cycle. Written informed consent was obtained from each patient. Transthoracic echocardiography was performed for all participants, demonstrating normal left ventricular systolic and diastolic function, with no evidence of wall motion abnormalities or significant valvular heart disease. All patients were referred to ETT for chest discomfort or angina-equivalent symptoms by their primary caretakers. Patients with high-risk characteristics, unstable symptoms, known CAD, uncontrolled hypertension, valvular heart disease, left ventricular hypertrophy, arrhythmia, heart failure, cancer, stage ≥3 kidney failure, abnormal systolic or diastolic function, wall motion abnormalities, pre-existing ECG abnormalities (e.g., left bundle branch block or ST abnormalities) that could confound ETT interpretation, liver failure, pregnancy, psychiatric illness, hormonal therapy or oral contraceptives, pericarditis, myocarditis, or recent infection were excluded. Women with irregular menstrual cycles, breakthrough bleeding, spotting, recent gynecological examinations, or any ongoing gynecological treatment that could disrupt the menstrual cycle were excluded from the study. All participants underwent a cardiovascular risk assessment, including an evaluation of hypertension, diabetes mellitus, smoking status, hyperlipidemia, family history of CAD, and medication use [13].

**Figure 1:**
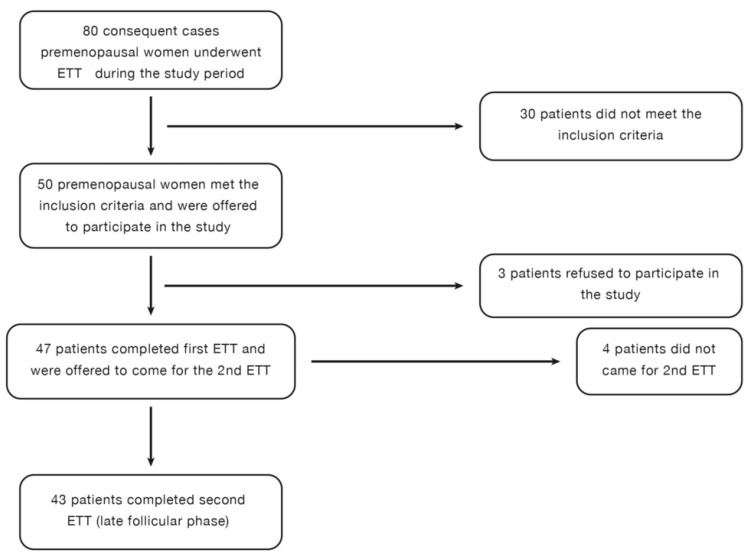
Study flowchart (ETT: exercise treadmill test).

The early and late follicular phases were determined based on patient history. The first ETT was scheduled for the third day of menstrual bleeding while bleeding was still ongoing. The second ETT was performed three days after the cessation of menstrual bleeding. Hormone levels were measured before each ETT to confirm the menstrual cycle phase. Each participant underwent two consecutive ETTs during the early and late follicular phases (Figure 1).

Blood samples were collected 30 min before the ETT to measure follicle-stimulating hormone (FSH), luteinizing hormone (LH), estrogen, progesterone, and high-sensitivity cardiac troponin (hs-cTnT). After the test, patients were allowed to rest for 15 min, after which a second blood sample was collected to measure hs-cTn levels post-ETT.

## 4. Exercise Treadmill Test Protocol

ETTs were performed using the standard Bruce protocol with the GE Healthcare T2100-ST Treadmill & CASE 6.73 Stress System [14].

The primary endpoint for comparison was the ST/HR index during ETT. Secondary endpoints included the ST/HR slope, ST segment depression, heart rate (HR) and blood pressure (BP) response, exercise capacity, and hs-cTnT levels.

Medications that could suppress the HR response, including beta-blockers, diltiazem, verapamil, or digoxin, were withheld 24 h before the ETT. Echocardiography was performed before the ETT. The Bruce protocol was followed using the GE Healthcare T2100-ST Treadmill & CASE 6.73 Stress System. The test was terminated once the target HR was achieved. The target HR was defined as >85% of the maximum predicted HR (220-age in years). Maximum, submaximum, and inadequate effort levels were defined as follows: HR > 95%, 85–95%, and <85% of the predicted maximum HR.

The ETT was prematurely terminated if the patient developed chest pain, ST elevation, significant ST depression (≥2 mm), arrhythmias, hypotension, or was unable to continue the test. All ETT-related data were recorded and analyzed using the GE Healthcare T2100-ST Treadmill & CASE 6.73 Stress System. ETT was conducted between 9:00 AM and 11:00 AM by the same technician.

## 5. Interpretation of ETT Results

The primary finding in ETT was ST segment deviation from the baseline. The ST segment was evaluated 60–80 ms after the J point. A positive ECG result was defined as ≥1 mm (0.1 mV) horizontal or downsloping ST depression in at least three consecutive beats. The presence of chest pain or an inadequate hemodynamic response during the ETT was considered a clinically positive ETT result. Tests that did not meet these criteria but showed ST segment changes or had low diagnostic value due to baseline ECG abnormalities were classified as indeterminate ETT results [15,16,17].

Automated measurements and calculations were recorded by the GE Healthcare T2100-ST Treadmill & CASE 6.73 Stress System. The ETT results were independently evaluated by two cardiologists and categorized as positive, indeterminate, or negative. Hs-cTn levels were measured before and 15 min after the ETT. Patients with positive ETT results were referred to their cardiologists for further evaluation.

The study’s primary endpoint was the ST/HR index. ST/HR slope and ST/HR index values were automatically reported by the GE Healthcare T2100-ST Treadmill & CASE 6.73 Stress System. Secondary endpoints included ST/HR slope, maximum ST segment depression (mm), HR and BP response to exercise, maximum exercise capacity, and change in hs-cTn levels post-ETT.

## 6. Hormone and hs-cTnT Levels

Blood samples (2 mL) were collected before and after each ETT for biochemical analysis. Plasma and serum were separated and stored at −80 °C until analysis. Hs-cTnT and hormone levels were measured using Roche Elecsys kits and ECLIA Cobas-E immunoassay analyzers, following the manufacturer’s protocol. The upper reference limit (URL) for hs-cTn was 14 ng/L, as specified by the manufacturer. All samples were taken into BD Vacutainer SST II Advance (Becton Dickinson, Franklin Lakes, NJ, USA) serum gel separator tubes (SSTs). After centrifugation at room temperature at 1500× *g* for 10 min, the tests were analyzed within 8 h after thawing. All hormone tests were measured using a fully automated chemiluminescent immunoassay using a Cobas e801 analyzer (Elecsys^®^, Cobas 8000, e801 immunoanalyzer; Roche Diagnostics, Mannheim, Germany) according to the manufacturer’s instructions. The tests were performed in an accredited laboratory. Reference values, analytical sensitivity, intra-assay coefficient of variation (CV) (min–max), and inter-assay CV (min–max) were periodically controlled through internal and external audits.

## 7. Statistical Analysis

All statistical analyses were performed using SPSS 25.0 for Windows (SPSS Inc., Chicago, IL, USA). The sample size was determined using the G*Power program, based on the expected effect size, type I error (5%), and study power (80%). For the assumed effect size of 0.5, the estimated sample size was calculated to be 34. The Kolmogorov–Smirnov or Shapiro–Wilk test was used to assess the normality of data distribution. Normally distributed numerical data were expressed as mean ± SD. Skewed numerical data were expressed as median (min–max) or interquartile range (IQR). Categorical variables were expressed as percentages (%). Categorical data were compared using the Chi-square test. Comparisons of numerical data before and after menstruation were performed using the paired *t*-test or Wilcoxon test, depending on data distribution. A two-sided *p*-value < 0.05 was considered statistically significant.

## 8. Results

A total of 80 premenopausal women (aged 20–55 years) were referred for ETT during the study period. After screening, 50 premenopausal women met the inclusion criteria (Figure 1). Among them, 47 patients agreed to participate, and 43 patients completed both ETTs. The study flowchart is presented in Figure 1. Cardiovascular risk factors and demographic variables of the study participants are presented in Table 1. The Framingham Risk Score was used to estimate the 10-year cardiovascular risk of the patients. The median 10-year risk of developing coronary heart disease was categorized as low (<10%) in the study group. Hormones at the early and late follicular phases are shown in Table 2. As expected, the late follicular phase is characterized by significantly increased estrogen and LH levels (33 vs. 105 pg/mL, *p* < 0.001 and 6.7 vs. 10.4, *p* < 0.001, respectively) (Table 2). The early follicular phase sample was obtained during the first two days of menstrual bleeding. No significant differences were detected in FSH and progesterone levels between the early and late follicular phases.

## 9. Exercise Treadmill Test Results

Comparisons of the exercise treadmill test (ETT) parameters are presented in Table 3. The blood pressure (BP) response during the first phase of ETT was significantly higher in the early follicular phase than in the late follicular phase. However, the average metabolic equivalent of task (METS) score and exercise duration were similar between the two phases (Table 3). The study endpoints, including ST/HR index, ST/HR hysteresis, and ST/HR slope, were comparable between the early and late follicular phases. The amount of ST depression at peak exercise and maximum ST depression during ETT were reported separately. The D2 lead demonstrated the maximum ST depression in both the early and late follicular phases, accounting for 57.1% and 53.1% of cases, respectively. The D3 lead showed maximum ST depression in 28.6% and 20.4% of early and late follicular phases, respectively (Table 4). Two cardiologists independently reviewed the ETT results. Early follicular phase ETTs were interpreted as indeterminate or positive in 11 and 10 versus 9 and 9 patients by the two cardiologists, respectively. Late follicular phase ETTs were classified as indeterminate or positive in seven and six versus eight and eight patients, respectively. There was fair agreement between the cardiologists (Kappa measure of agreement: 0.330, *p* = 0.003). No complications were observed during or after the ETTs. One patient experienced atypical chest pain that persisted post-test but resolved during recovery. This patient did not experience any cardiovascular events during a one-year follow-up. Another patient had a positive high-sensitivity cardiac troponin (hs-cTn) result (18 ng/L) after the ETT, despite being asymptomatic and having a normal echocardiography. This patient also did not experience any cardiovascular events within one year.

## 10. High-Sensitivity Cardiac Troponin (hs-cTn) Levels Before and After ETT

**Figure 2:**
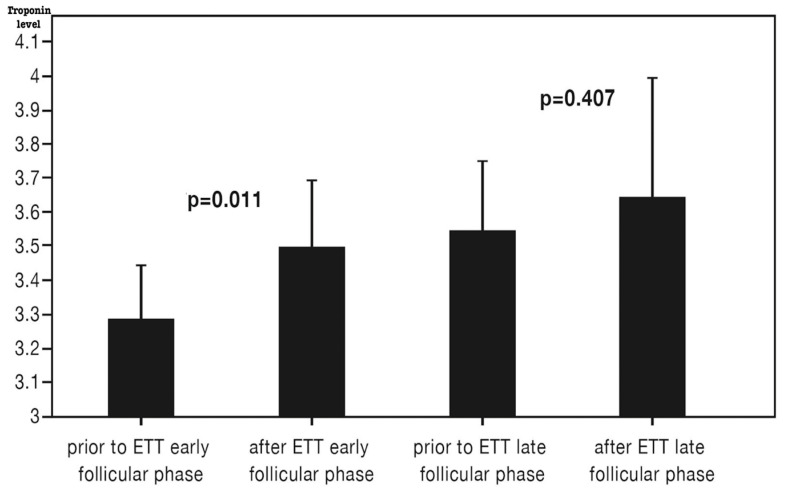
High-sensitivity cardiac troponin T levels before and after the exercise treadmill test at the early and late follicular phases (ETT: exercise treadmill test).

Hs-cTnT levels were measured before and after ETT in both the early and late follicular phases (Table 2, Figure 2). The median hs-cTnT level was below the detectable range (<3 ng/L). Baseline hs-cTnT before ETT was significantly higher in the late follicular phase compared to the early follicular phase (3.6 ± 1.4 vs. 3.3 ± 1.1, *p* = 0.013). A significant increase in hs-cTnT was observed following ETT in the early follicular phase, whereas no significant change was noted in the late follicular phase (Figure 2). However, hs-cTnT levels 15 min post-ETT were similar between the two phases (3.5 ± 1.3 vs. 3.6 ± 2.5, *p* = 0.794).

Sixteen patients underwent stress echocardiography, all of which were negative for ischemia. Three patients underwent computed tomography (CT) coronary angiography, and two underwent conventional coronary angiography for further evaluation. None of the angiographic assessments revealed significant (>50%) coronary obstruction.

All patients were followed up for 1 year. No cardiac events or mortality were observed. Eight patients presented to the emergency room (ER) with chest pain; hs-cTnT levels were negative in all cases. One patient was admitted to the ER due to an asthma attack, and another following a suicide attempt.

## 11. Discussion

Prior studies have suggested that ETT has a poor diagnostic performance in women [18,19,20,21]. In an observational study, the false-positive ETT result (defined as a positive ETT with angiographically normal coronary arteries or <50% stenosis) was 8% in men and 67% in women, respectively [20]. Being female by itself is considered to be a risk factor for a ‘false-positive’ ETT [18,19,20,21]. The causes of false-positive test results in women are poorly understood.

Our observations confirm that false-positive ETT results are common in premenopausal women, and that the menstrual cycle phase (early versus late follicular phase) does not affect the ETT results.

Hormones play an important role in the CAD pathophysiology. Hormones are involved in the functional and structural abnormalities of the coronary circulation in young women. Menopause increases the CAD risk in women. Longitudinal studies indicate that premenopausal women with increased cardiovascular risk often experience menopause early and suffer from their first CVD event before 35 years of age [22,23,24].

The effects of endogenous and exogenous estrogen on coronary ischemia are complex and extend beyond the scope of this paper. This study aims to assess the relationship between the endogenous estrogen production and the false-positive response to ETT in premenopausal women. Studies report contradictory findings on the vascular effects of estrogen. Estrogen has both vasodilatory and vasoconstrictor effects on the vasculature. Experimental studies indicate estrogen improves vascular function by vasodilation and lowering blood pressure [25].

On the other hand, studies have also suggested that estrogen can cause vasoconstriction by increasing catecholamine sensitivity, the renin–angiotensin–aldosterone system, and α-adrenergic affinity [26,27,28].

A prior study with a similar design to ours compared the two consecutive ETT results before and after exogenous oral estrogen therapy in postmenopausal women. Comparison of the ETT results demonstrates that 20% of postmenopausal women with normal stress echocardiography develop false-positive ETT results after the start of oral estrogen treatment [29].

In our study, we do not observe differences in ETT results. The mechanisms of false-positive ETT after estrogen remain poorly understood. The postmenopausal women have more frequent cardiovascular risk factors and possible endothelial dysfunction compared to the premenopausal women. Therefore, it is plausible that the neuro-hormonal and vascular response to estrogen can differ in the presence of endothelial dysfunction and cardiovascular risk.

Estrogen and digoxin have similar chemical structures; estrogen can induce false-positive ST segment changes with a similar mechanism to digoxin [30].

In a previous study, it was found that the frequency of ischemic episodes changed with the changing estrogen levels in premenopausal women [31,32]. The study findings suggest that the early follicular phase displays significantly higher BP response to ETT compared to the late follicular phase. Exaggerated BP response at ETT is associated with the risk of CAD [33,34]. The mechanisms for BP differences between the early and late follicular phases are not clear. The neuro-hormonal and vascular response to estrogen can potentially explain the differences in BP in response to exercise between different phases of the menstrual cycle.

Baseline hs-cTnT levels were higher in the late than the early follicular phase. It remains to be elucidated if hormonal changes in the menstrual cycle influence the release kinetics of hs-cTnT in premenopausal women. Biological and analytical variations exist in hs-cTn assays. The Academy of the American Association for Clinical Chemistry indicates that gender, age, and ethnicity of the reference population can affect the baseline cTnT levels [35]. Our observations imply that hormonal variations in premenopausal women contribute to the biological variations in hs-cTnT. The clinical significance of ETT-induced hs-cTn leak remains unknown.

Release of cTn after ETT has been observed after prolonged exercise [36]. Whether changes in troponin below the upper reference limit carry a risk for CVD is still a matter of debate, and new studies are needed [37].

The lymphatic muscle shares several structural and electrophysiological properties of the cardiac muscle. The release of cTn after ETT is likely not from the heart but from the cardiac cells incorporated in the lymphangion [38,39]. Future studies are needed to elucidate he hormonal effects of estrogen on the lymphangion and volume status (edema).

## 12. Limitations

We acknowledge that this study has several limitations. The sample size is small and cross-sectional. The study population has a low risk for CAD. The study population does not represent the entire premenopausal population. The diagnostic and prognostic forecast value of ETT will also change according to the patient population. We cannot rule out the possibility of variation in ETT performance between the other phases of the menstrual cycle (i.e., ovulation or luteal phases). Understanding the role of hormonal changes, including estrogen, in exercise performance is complex. Several paradoxical observations have been reported about the associations between hormone levels and CAD risk in women. Estrogen may have a dual role in the coronary circulation and functional capacity [30].

Patient phenotype can significantly affect the ETT results. We examine a representative sample of premenopausal women from Istanbul, Turkey. Finally, hs-cTn levels are obtained 15 min after ETT. The half-life of cTnT in the circulation is 120 min [40]. Follow-up cTnT assessments could have demonstrated further changes in cTn after ETT. Serial measurements of cTn within the same individual could have helped reduce the inherent biological variability.

## 13. Conclusions

The role of hormonal variations in the poor performance of ETT in young women is poorly understood. This study tested the hypothesis that the phase of the menstrual cycle affects the ETT parameters in premenopausal women. False-positive ETT results were common in premenopausal women and the menstrual cycle phase (early versus late follicular phase) did not affect the ETT results. We observed differences in secondary endpoints of BP response and hs-cTnT release after ETT. The consideration of estrogen and hormonal status in evaluating the diagnostic test results can improve our understanding of cardiovascular disease in women.

## Figures and Tables

**Table 1:** The demographic variables of the study participants. CAD: coronary artery disease, LDL: low-density lipoprotein, HDL: high-density lipoprotein, IQR: interquartile range, %: percentage.

Demographic Variables	Median (IQR)
History of hypertension (%)	10 (20%)
Diabetes mellitus (%)	5 (10%)
History of smoking (%)	24 (48%)
History of hyperlipidemia (%)	12 (24%)
Family history of CAD (%)	26 (52%)
Age (years)	38 (11)
Fasting blood glucose (mg/dL)	92 (15)
Serum creatinine (mg/dL)	0.7 (0.1)
LDL cholesterol (mg/dL)	110 (40)
HDL cholesterol (mg/dL)	55 (18)
Triglycerides (mg/dL)	89 (81)
Total cholesterol (mg/dL)	186 (32)
Non-HDL (mg/dL)	127 (40)
Hemoglobin (g/dL)	12.7 (1.3)
Hematocrit %	38 (3.8)
Body mass index (kg/m^2^)	26.1 (11)

**Table 2:** The hormonal and high-sensitivity cardiac troponin T (hs-cTnT) differences at the early and late follicular phases. Results confirm that estrogen and luteinizing hormone increase at the late follicular phase. Estrogen is the dominant hormone until the beginning of the luteal phase. The Wilcoxon signed-rank test is used to compare the non-parametric paired samples. Median (interquartile range). Statistical test: Wilcoxon test; significance: *p* < 0.05. ETT: exercise treadmill test, hs-cTnT: high-sensitivity cardiac troponin T. Median and interquartile range are displayed for estrogen, follicle-stimulating hormone, luteinizing hormone, and progesterone. Mean and standard deviation (SD) are displayed for hs-cTnT.

	Early Follicular Phase	Late Follicular Phase	z	*p*
Estrogen (pg/mL)	33 (41)	105 (161)	−4.450	<0.001
Follicle-stimulating hormone (IU/mL)	6.9 (6.2)	5.7 (6.7)	−1.739	0.082
Luteinizing hormone (IU/L)	6.7 (4.1)	10.4 (9.4)	−4.376	<0.001
Progesterone (ng/mL)	0.25 (0.2)	0.18 (0.6)	−0.632	0.528
hs-cTnT before ETT (ng/L)	3.3 (1.1)	3.6 (1.4)	−2.485	0.013
hs-cTnT after ETT (ng/L)	3.5 (1.3)	3.6 (2.5)	−0.794	0.794

**Table 3:** Hemodynamic response parameters of the exercise treadmill test. Systolic and diastolic blood pressure at stage 1 are higher in the early than in the late follicular phase. Data presented as mean (standard deviation). Statistical test: paired sample *t*-test; significance: *p* < 0.05. BP: blood pressure, mn: minute.

Variables	Early Follicular Phase	Late Follicular Phase	*p*	*t*-Score
Target heart rate (/mn)	180 (8)	180 (7)	0.808	0.244
Baseline heart rate (/mn)	84 (12)	84 (12)	0.806	0.248
Baseline systolic BP (mmHg)	121 (10)	122 (12)	0.742	−0.332
Baseline diastolic BP (mmHg)	78 (12)	77 (9)	0.669	0.430
Heart rate stage-1 (/mn)	121 (16)	119 (18)	0.32	1.006
Heart rate stage-2 (/mn)	136 (25)	138 (15)	0.561	−0.586
Heart rate stage-3 (/mn)	155 (14)	154 (15)	0.367	0.913
Systolic BP stage-1 (mmHg)	142 (21)	132 (19)	0.002	3.23
Diastolic BP stage-1 (mmHg)	79 (13)	75 (13)	0.037	2.154
Systolic BP stage-2 (mmHg)	155 (23)	152 (22)	0.377	0.893
Diastolic BP stage-2 (mmHg)	77 (15)	76 (14)	0.648	0.460
Metabolic equivalents (METs)	10.0 (1.9)	10.5 (1.2)	0.111	−1.631
Exercise time (mn)	7.4 (0.7)	7.6 (0.7)	0.213	−1.263
Heart rate recovery (/mn)	100 (14)	100 (14)	0.868	0.167
Systolic BP recovery (mmHg)	132 (16)	131 (17)	0.650	23.289
Diastolic BP recovery (mmHg)	76 (12)	75 (12)	0.661	−16.304

**Table 4:** Electrocardiographic parameters of the exercise treadmill test. Maximal ST segment deviation in lead 2 is higher (regardless of upsloping, downsloping, or horizontal) in the late than the early follicular phase. Data presented as mean (standard deviation). Statistical test: paired sample *t*-test; significance: *p* < 0.05. SD: standard deviation, ST: ST segment, mm: millimeter, ms: millisecond.

Variables	Early Follicular Phase	Late Follicular Phase	*p*	*t*-Score
Max ST segment change (mm)	0.13 (0.08)	0.15 (0.08)	0.062	−1.362
ST/heart rate index	1.2 (0.82)	1.3 (0.59)	0.521	−0.425
ST/heart rate slope	1.8 (1.20)	1.7 (0.50)	0.414	0.265
Heart rate reserve %	73.6 (16.4)	74.9 (12.4)	0.571	0.570
ST/heart rate hysteresis	0.01 (0.01)	0.02 (0.02)	0.785	−0.903
QRS duration (ms)	85.5 (6.5)	84.5 (8.2)	0.413	0.826
ST deviation at peak exercise DI (mm)	0.028 (0.023)	0.028 (0.031)	0.88	0.042
ST deviation at peak exercise DII (mm)	0.083 (0.06)	0.100 (0.061)	0.034	−1.954
ST deviation at peak exercise DIII (mm)	0.084 (0.055)	0.093 (0.057)	0.349	−1.084
Max ST deviation DI (mm)	0.034 (0.034)	0.051 (0.060)	0.075	−1.826
Max ST deviation DII (mm)	0.113 (0.094)	0.122 (0.083)	0.512	−0.662
Max ST deviation DIII (mm)	0.103 (0.085)	0.118 (0.076)	0.26	−1.141
Max ST deviation AVR (mm)	0.063 (0.056)	0.078 (0.059)	0.156	−1.444
Max ST deviation AVL (mm)	0.059 (0.043)	0.067 (0.060)	0.428	−0.800
Max ST deviation AVF (mm)	0.107 (0.088)	0.117 (0.073)	0.394	−0.861
Max ST deviation V1 (mm)	0.061 (0.049)	0.055 (0.042)	0.362	0.923
Max ST deviation V2 (mm)	0.054 (0.034)	0.055 (0.038)	0.801	−0.253
Max ST deviation V3 (mm)	0.061 (0.053)	0.061 (0.048)	0.953	0.059
Max ST deviation V4 (mm)	0.069 (0.061)	0.065 (0.059)	0.646	0.463
Max ST deviation V5 (mm)	0.077 (0.064)	0.074 (0.056)	0.735	0.341
Max ST deviation V6 (mm)	0.076 (0.060)	0.076 (0.052)	0.973	0.034

## Data Availability

The original contributions presented in this study are included in the article. Further inquiries can be directed to the corresponding authors.
